# Magnetic resonance imaging T1 mapping of the liver, pancreas and spleen in children

**DOI:** 10.1007/s00261-024-04428-z

**Published:** 2024-06-26

**Authors:** Pradipta Debnath, Jean A. Tkach, Michelle Saad, David S. Vitale, Maisam Abu-El-Haija, Andrew T. Trout

**Affiliations:** 1 Department of Radiology, Cincinnati Children’s Hospital Medical Center, 3333 Burnet Ave, Kasota Building MLC 5031, Cincinnati, OH 45229 USA; 2 Department of Radiology, University of Cincinnati College of Medicine, Cincinnati, OH USA; 3 Department of Pediatrics, University of Cincinnati College of Medicine, Cincinnati, OH USA; 4 Division of Gastroenterology, Hepatology and Nutrition, Cincinnati Children’s Hospital Medical Center, Cincinnati, OH USA

**Keywords:** MRI, Pancreatitis, Pediatrics, Steatosis, T1 mapping

## Abstract

**Purpose:**

To characterize T1 relaxation times of the pancreas, liver, and spleen in children with and without abdominal pathology.

**Methods:**

This retrospective study included pediatric patients (< 18-years-old). T1 mapping was performed with a Modified Look-Locker Inversion Recovery sequence. Patients were grouped based on review of imaging reports and electronic medical records. The Kruskal–Wallis test with Dunn’s multiple comparison was used to compare groups.

**Results:**

220 participants were included (mean age: 11.4 ± 4.2 years (1.5 T); 10.9 ± 4.5 years (3 T)). Pancreas T1 (msec) was significantly different between subgroups at 1.5 T (p < 0.0001). Significant pairwise differences included: normal (median: 583; IQR: 561–654) vs. acute pancreatitis (731; 632–945; p = 0.0024), normal vs. chronic pancreatitis (700; 643–863; p = 0.0013), and normal vs. acute + chronic pancreatitis (1020; 897–1099; p < 0.0001). Pancreas T1 was also significantly different between subgroups at 3 T (p < 0.0001). Significant pairwise differences included: normal (779; 753–851) vs. acute pancreatitis (1087; 910–1259; p = 0.0012), and normal vs. acute + chronic pancreatitis (1226; 1025–1367; p < 0.0001).

Liver T1 was significantly different between subgroups only at 3 T (p = 0.0011) with pairwise differences between normal (818, 788–819) vs. steatotic (959; 848–997; p = 0.0017) and normal vs. other liver disease (882; 831–904; p = 0.0455). Liver T1 was weakly correlated with liver fat fraction at 1.5 T (r = 0.39; 0.24–0.52; p < 0.0001) and moderately correlated at 3 T (r = 0.64; 0.49–0.76; p < 0.0001).

There were no significant differences in splenic T1 relaxation times between subgroups.

**Conclusion:**

Pancreas T1 relaxation times are higher at 1.5 T and 3 T in children with pancreatitis and liver T1 relaxation times are higher in children with steatotic and non-steatotic chronic liver disease at 3 T.

**Supplementary Information:**

The online version contains supplementary material available at 10.1007/s00261-024-04428-z.

## Introduction

MRI relaxometry is a method used to quantitatively characterize MRI findings. Various relaxometry measures have been explored as potential quantitative MR imaging biomarkers [1, 2]. T1 relaxometry (T1 mapping) measures how fast nuclear spin magnetization returns to its equilibrium state following excitation by a radiofrequency pulse and can be performed without (native T1) or following intravenous administration of gadolinium-based contrast material [3]. T1 relaxometry has been used to quantify myocardial fibrosis and has been explored as a putative measure of liver fibrosis and inflammation [4–9].

T1 mapping can theoretically be applied to any organ in the body. In the abdomen, in addition to the liver, T1 mapping of the pancreas has been shown to identify fat deposition, chronic pancreatitis and autoimmune pancreatitis in adults [10–12]. Also in adults, T1 mapping of the spleen has been shown to be useful in the assessment of portal hypertension and liver fibrosis [13, 14]. To date, there is a relative paucity of literature regarding T1 mapping of the pancreas, liver and spleen in children and there are limited data regarding normal T1 values in these organs in children. Specifically, Gilligan et al. previously reported T1 relaxation times of liver, pancreas and spleen in a small sample (n = 32) of healthy children [15].

The purpose of this study was to characterize T1 relaxation times of the pancreas, liver and spleen in a large sample of children with and without abdominal pathology.

## Methods

This is a retrospective study conducted at Cincinnati Children's Hospital Medical Center, a tertiary, academic pediatric center. The study protocol was approved by the institutional review board with a waiver of documentation of written informed consent. All study activities were compliant with the Health Insurance Portability and Accountability Act.

**Fig. 1:**
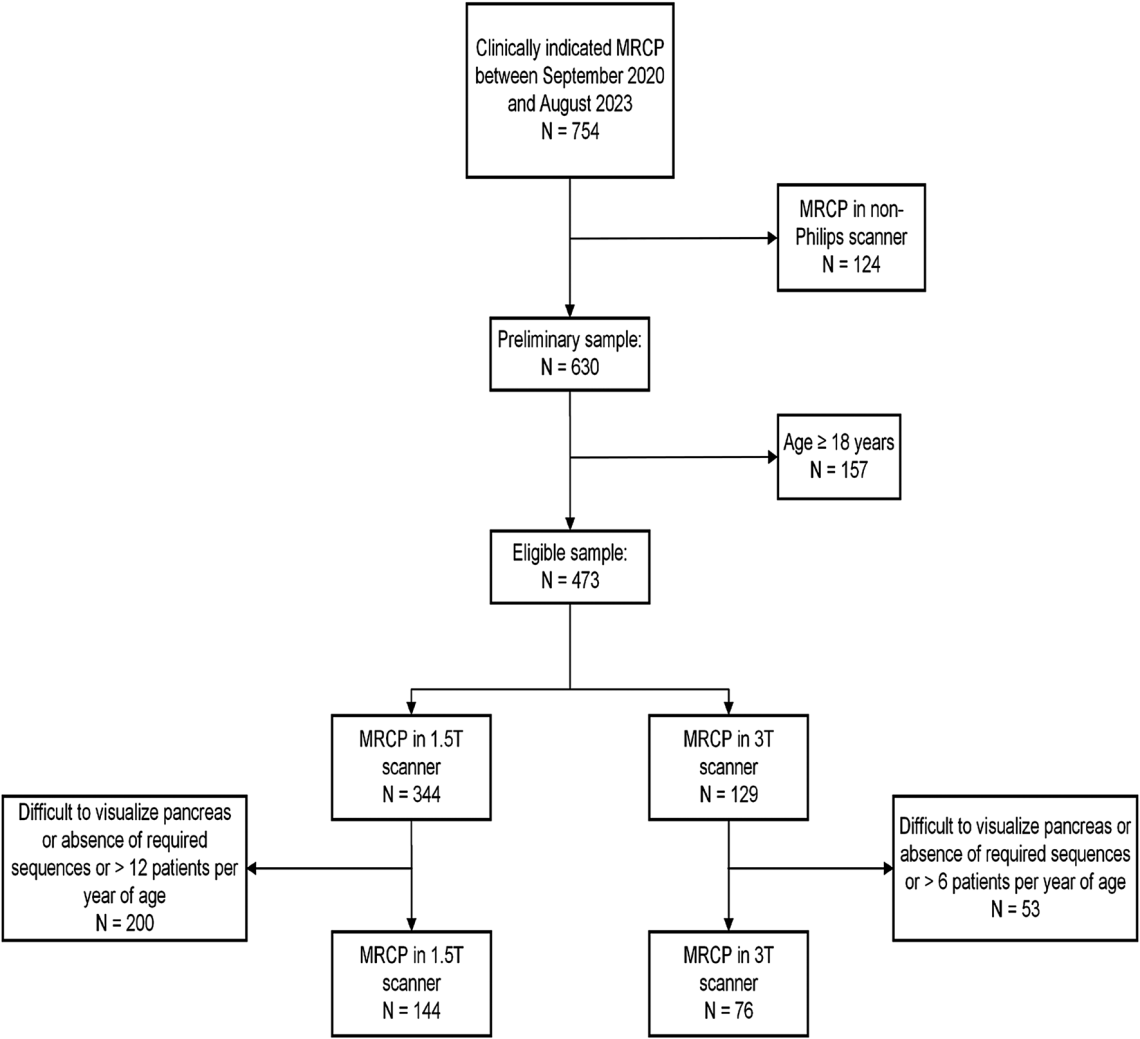
Flow diagram shows study sample selection

At our institution, clinically performed MR cholangiopancreatography (MRCP) examinations routinely include acquisition of T1 mapping data. A research fellow (P.D.) used an imaging report search engine (Illuminate InSight v4.3, Softek Illuminate) to identify patients who had undergone a clinically indicated MRCP examination between January 1, 2020, and August 31, 2023. Because of potential technical differences in implementation of T1 mapping, we limited inclusion to examinations performed on Philips MRI scanners. We then excluded patients who were 18 years of age or older on the day of their MRCP examination. Among the remaining patients, we sub-categorized our patient sample into those scanned on 1.5 T versus 3 T scanners. Finally, to ensure a relatively even distribution of ages, we limited the number of included examinations with a visible and measurable pancreas per year of age to 12 for the 1.5 T scanner and 6 per year of age on the 3 T scanner based on selection of consecutive examinations. The patient selection process is summarized in Fig. 1.

### MRI examinations

All included clinical MRI examinations had been performed on one of the following three MRI scanner platforms: Philips Ingenia 1.5 T, Philips Ingenia 3 T, and Philips Ambition 1.5 T (Philips Healthcare, Best, The Netherlands). Routine T1 mapping as part of clinical MRCP examination protocols at our institution is accomplished using an Electrocardiogram (ECG)-triggered two-dimensional Modified Look-Locker Inversion Recovery (MOLLI) sequence implemented as a (5 s(3 s)3 s) scheme using a balanced steady state free precession (bSSFP). An MRI system simulated ECG signal of 60 beats per minute was used to trigger the acquisition. Four axial slices, positioned to cover the pancreas, and thus also including portions of the liver and spleen, were acquired as one slice per 11 s end-expiration breath hold. Sequence parameters were as follows: TR, 3.2 ms; TE, 1.32 ms; 16 TIs at 122, 347, 572,797, 1022, 1247, 1472, 1697, 1922, 2147, 2372, 2597, 2822,3047, 3272, and 3497 ms; flip angle, 8 degrees; FOV, 380 × 309 mm2; matrix, 192 × 125; slice thickness, 5 mm; acquisition time, 11 s/slice location.

T1 relaxation parametric maps, with 95% confidence maps overlaid, were generated immediately on the scanner console using the vendor’s product software and then exported to the picture archiving and communication system (PACS) (Merge PACS, version 7.2.0.157991, Merge Healthcare). The T1 estimation was based on a single compartment model, i.e., assuming that the signal evolution within a given voxel is described by a single T1 relaxation time.

Clinical MRCP examinations also included the routine acquisition of proton density fat fraction (PDFF) images. These images are acquired using the Philips product three dimensional mDixon Quant® sequence over an independent single breath hold (of ~ 5–13 s duration). As with the T1 parametric maps, the PDFF parametric maps were generated immediately on the scanner console using the vendor’s product software. However, unlike for the T1 parametric maps, the vendor software does not generate 95% confidence maps overlays for the PDFF parametric maps. Once generated, the PDFF parametric maps were exported to the PACS.

### Image analysis

**Fig. 2:**
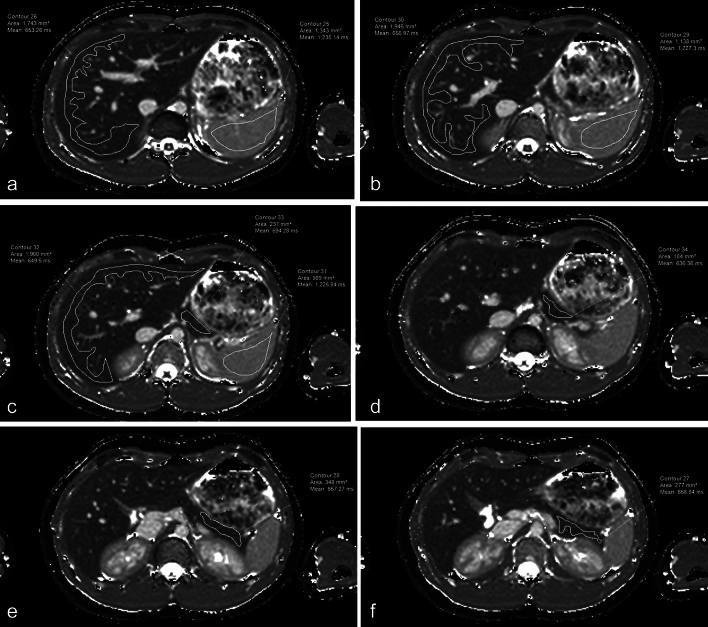
Six images (**A**–**F**) from a T1 parametric map acquired at 1.5 T in a healthy (no disease) 14-year-old girl. Regions of interest are drawn in the liver, pancreas and spleen. Regions of interest were drawn to be as large as possible while avoiding large vessels, any lesion, and areas of low-confidence data defined by scanner-generated confidence maps

The same research fellow (P.D.) used a vendor-neutral post-processing platform (IntelliSpace, Philips Healthcare) to record T1 estimates for the liver, pancreas and spleen. Freehand regions of interest (ROIs) were drawn on the T1 parametric maps to include as much of the relevant organ as possible and excluding all lesions, large vessels and areas of artifact while staying within the 95% confidence maps (Fig. 2). For each patient, a maximum of three ROIs were drawn for liver and a maximum of three ROIs were drawn for the spleen. Each of the three ROIs were drawn on images acquired at different slice locations. For the pancreas, due to the relatively smaller areas and appreciable spatial heterogeneity of the acceptable 95% confidence regions, a maximum of eight ROIs were drawn, with more than one ROI drawn on images acquired at the same slice location as necessary to maximize measurement area.

Liver PDFF was measured by the same research fellow by drawing a separate smoothed contour ROI in the right hepatic lobe on the scanner generated PDFF parametric map. A PDFF greater than 5% was considered to reflect steatosis.

All ROIs were reviewed by a board-certified pediatric radiologist with more than 10 years of post-fellowship experience (A.T.T.). Once confirmed, T1 relaxation time estimates were reported as a mean weighted by their respective ROI areas calculated as:

$$Mean\ T1\ relaxation\ time = \frac{\left({Area}_{1st\ ROI}\ *\ {T1\ relaxation\ time}_{1st\ ROI}\right)}{\left(sum\ of\ areas\ of\ n\ ROIs\right)} +\dots +\frac{\left({Area}_{nth\ ROI}\ *\ {T1\ relaxation\ time}_{nth\ ROI}\right)}{\left(sum\ of\ areas\ of\ n\ ROIs\right)}$$

### Medical record review

For all included patients, electronic health records (Epic; Verona, WI) were reviewed to record demographics, anthropometric data, and any relevant health conditions (involving the liver, spleen, pancreas, inflammatory bowel disease). Additionally, relevant laboratory values were recorded which included alanine aminotransferase, aspartate aminotransferase, alkaline phosphatase, gamma-glutamyl transferase, amylase and lipase within 24 h of imaging date. The radiology reports were also reviewed to identify the indications and imaging findings relevant to the liver, pancreas (including acute pancreatitis and chronic pancreatitis), and spleen.

### Patient grouping

**Fig. 3:**
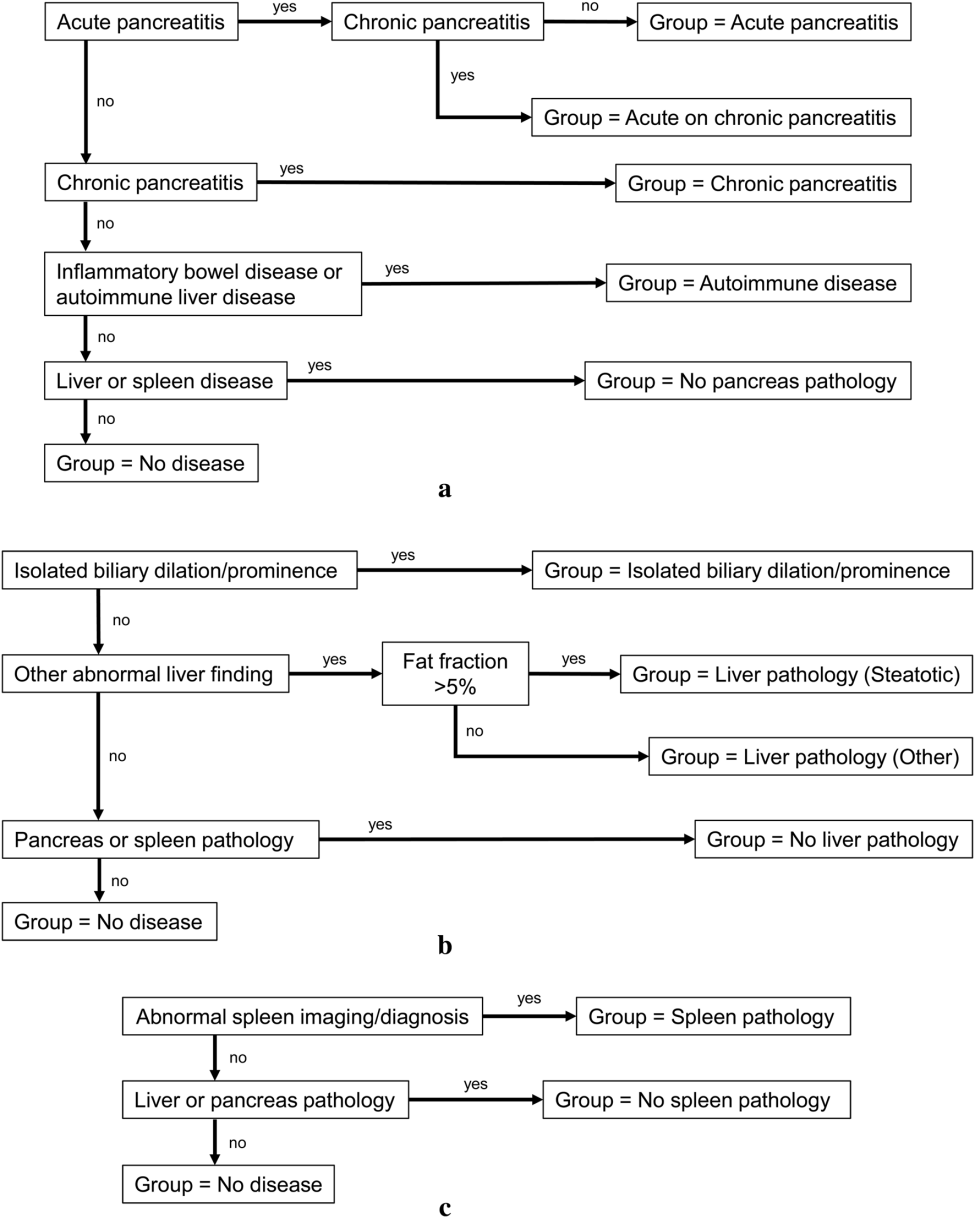
Patient grouping scheme for **A** Pancreas, **B** Liver, **C** Spleen diagnoses

Patient subgroups were defined based on review of imaging reports, electronic medical records and laboratory values. Grouping strategies are summarized in Fig. 3. Acute pancreatitis was defined by meeting at least two of three International Study Group of Pediatric Pancreatitis (INSPPIRE) diagnostic criteria (imaging findings, lipase ≥ 3 × the upper limit, or abdominal pain) [16]. Chronic pancreatitis was defined also based on the INSPPIRE criteria which included imaging findings (ductal and/or parenchymal changes) plus one of the following: abdominal pain in the pancreatic region, pancreatic exocrine insufficiency or pancreatic endocrine insufficiency [16]. The no pancreas pathology group included patients with no evidence of pancreatic disease but with abnormalities of the liver or spleen distinguishing this group from the no disease group who had no evidence of disease in the pancreas, liver, or spleen. Liver and spleen pathology was identified from imaging findings, medical record problem lists and laboratory values. Liver groupings included five or three groups (Fig. 3) with the 3-group scheme based on creating a combined no disease group including: Isolated biliary dilation/prominence, no liver pathology (but pancreas or liver pathology), no disease. Spleen grouping consisted of spleen pathology (e.g., splenomegaly, acquired aspenia), no spleen pathology (but liver or pancreas pathology) and no disease. Based on patient grouping, scatterplots were created to visualize the distribution of T1 relaxation time estimates by organ. Outliers in each group were reviewed by the same board-certified pediatric radiologist (A.T.T.), who was blinded at the time to group assignment, to confirm the quality of images, and to assess for imaging findings not included in the original report. Outliers were defined as follows: Pancreas: no disease or no pancreas pathology with T1 relaxation time greater than 800 ms (1.5 T) or 850 ms (3 T) and any case with pancreatitis (acute or chronic or both) with T1 relaxation time less than 600 ms (1.5 T) or 750 ms (3 T); Liver: no disease or no liver pathology with T1 time greater than 700 ms (1.5 T) or 900 ms (3 T) and any case with liver disease with T1 relaxation time less than 600 ms (1.5 T) or 850 ms (3 T). After review, outliers were annotated with an explanation if identifiable (e.g., excess iron or fat deposition) or patient grouping was changed if previously unidentified pathology was identified (e.g. segmental pancreatitis).

### Statistical analysis

Means and standardized deviations or medians and interquartile ranges (IQR) were used to summarize continuous data. Counts and percentages were used for categorical data. The Kruskal–Wallis test was used to compare T1 relaxation time estimates between subgroups with Dunn’s test used for pairwise comparisons. Pearson correlations were calculated between predictor and outcome variables and their strength of correlation was classified as follows: 0–0.19, very weak; 0.2–0.39, weak; 0.40–0.59, moderate; 0.60–0.79, strong; and 0.80–1.0, very strong [17]. A p value < 0.05 was considered to be statistically significant. Statistical analyses were performed using MedCalc Statistical Software version 22.009 (MedCalc Software Ltd., Ostend, Belgium) and GraphPad Prism software (version 9.5.0 for Windows).

## Results

Two hundred twenty patients were included in our study, 144 imaged at 1.5 T and 76 imaged at 3 T. For patients imaged at 1.5 T, the mean age was 11.4 ± 4.2 years and 52.8% (76/144) were female. For patients imaged at 3 T, the mean age was 10.9 ± 4.5 years and 50.0% (38/76) were female. Patient demographics and their grouping based on pancreas, liver and spleen findings are summarized in Table 1.

**Table 1:** Study sample demographics and diagnosis grouping (per the scheme in Fig. 3) for pancreas, liver and spleen for patients imaged on 1.5 T and 3 T MRI scanners. For liver grouping, two different schemes were tested, one with five categories and one with three categories. For the three-category grouping the no disease, no liver pathology, and isolated biliary dilation/prominence groups were combined into a single group

Variable	1.5 T scanners (n = 144) [n, %]	3 T scanners (n = 76) [n, %]
Age (Years)*	11.4 ± 4.2	10.9 ± 4.5
Sex	F = 76 (52.8%) M = 68 (47.2%)	F = 38 (50.0%) M = 38 (50.0%)
Indications for imaging	Pancreatitis = 79 (54.8%) Exocrine pancreatic insufficiency = 11 (7.6%) Choledochal cyst = 6 (4.2%) Biliary duct dilation = 5 (3.5%) Elevated liver enzymes = 5 (3.5%) Abdominal pain = 4 (2.8%) Autoimmune hepatitis = 4 (2.8%) Evaluate pancreas = 4 (2.8%) Other (each n < 4) = 26 (18.0%)	Pancreatitis = 53 (69.8%) Evaluate pancreas = 3 (3.9%) Abdominal pain = 2 (2.6%) Biliary duct dilation = 2 (2.6%) Evaluation for Total Pancreatectomy with Islet Autotransplantation = 2 (2.6%) Trauma = 2 (2.6%) Other (each n < 2) = 12 (15.9%)
Pancreas diagnosis grouping	Autoimmune disease = 9 (6.3%) AP = 18 (12.5%) AP + CP = 19 (13.2%) CP = 28 (19.4%) No pancreas pathology = 47 (32.6%) No disease = 23 (16.0%)	Autoimmune disease = 2 (2.6%) AP = 19 (25.0%) AP + CP = 15 (19.7%) CP = 15 (19.7%) No pancreas pathology = 13 (17.1%) No disease = 12 (15.9%)
Liver diagnosis grouping (5 categories)	Abnormal (steatotic) = 60 (41.7%) Abnormal (Other) = 21 (14.5%) Isolated biliary dilation/prominence = 7 (4.9%) No liver pathology = 33 (22.9%) No disease = 23 (16.0%)	Abnormal (steatotic) = 15 (19.7%) Abnormal (Other) = 23 (30.3%) Isolated biliary dilation/prominence = 2 (2.6%) No liver pathology = 25 (32.9%) No disease = 11 (14.5%)
Liver grouping (3 categories)	Abnormal (steatotic) = 60 (41.7%) Abnormal (Other) = 21 (14.5%) Isolated biliary dilation/prominence + No liver pathology + No disease = 63 (43.8%)	Abnormal (steatotic) = 15 (19.7%) Abnormal (Other) = 23 (30.3%) Isolated biliary dilation/prominence + No liver pathology + No disease = 38 (50.0%)
Spleen diagnosis grouping †	Abnormal = 14 (10.0%) No spleen pathology = 105 (75.0%) No disease = 21 (15.0%)	Abnormal = 17 (23.0%) No spleen pathology = 47 (63.5%) No disease = 10 (13.5%)

*F* female; *M* male; *AP* acute pancreatitis; *AP* + *CP* acute on chronic pancreatitis; *CP* chronic pancreatitis

^*^Results presented as means and standard deviation

^†^Only 140 (out of 144) had measurable spleen value at 1.5 T and 74 (out of 76) had measurable spleen value at 3 T

### Pancreas T1 relaxation time

Median T1 relaxation time in patients with no disease was 583 (IQR: 561 to 654) msec at 1.5 T and 779 (753 to 851) msec at 3 T. This was not significantly different (p > 0.999 for both) from patients with no pancreatic pathology [599 (560 to 649) msec at 1.5 T and 827 (797 to 904) msec at 3 T].

**Fig. 4:**
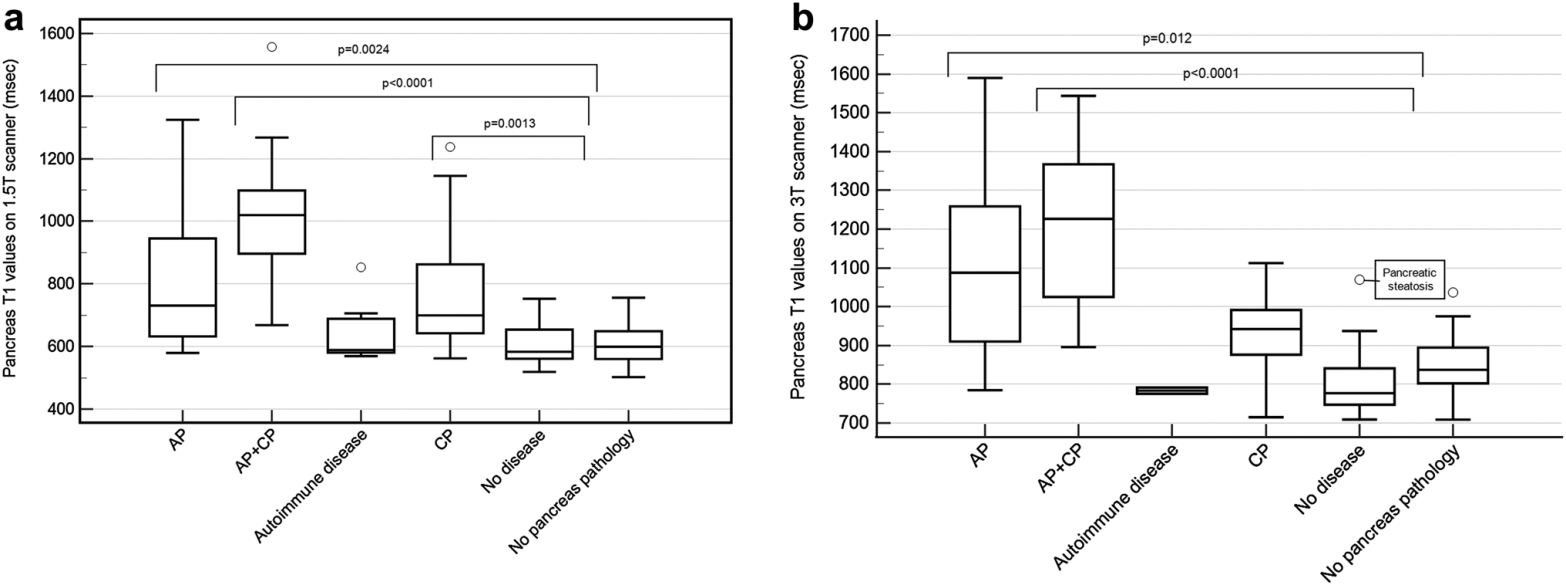
Tukey boxplots showing weighted (by region of interest size) T1 time estimates for pancreas (msec) at (**A**) 1.5 T and (**B**) 3 T. Groupings are based on diagnoses assigned according the scheme in Fig. 3 and include autoimmune disease, acute pancreatitis (AP), acute on chronic pancreatitis (AP + CP), chronic pancreatitis (CP), no pancreas pathology, and no disease (no pathology of any organ, including pancreas). Circles represent outliers and are accompanied by explanations (if applicable). Significant pairwise comparisons (if present) between the no disease group and other groups are shown with brackets along with their respective p-value

At 1.5 T, there were significant differences in T1 relaxation time estimates between pancreas subgroups (p < 0.0001, Fig. 4 and Table 2). Significant pairwise differences (msec) were present between patients with no disease and patients with: acute pancreatitis (731; 632 to 945 ms, p = 0.004), chronic pancreatitis (700; 643 to 863, p = 0.0013) and acute + chronic pancreatitis (1020; 897 to 1099, p < 0.0001). T1 relaxation time estimates for patients with acute + chronic pancreatitis were not significantly different from patients with acute pancreatis (p = 0.299) or chronic pancreatitis (p = 0.066).

**Table 2:** Summary statistics for pancreas, liver and spleen T1 relaxation time estimates based on diagnostic grouping at 1.5 T. Pairwise comparisons were performed using Dunn’s test if Kruskal–Wallis test was statistically significant

Organ	Diagnosis Group	T1 relaxation time Median (IQR) [msec]	Significant (p < 0.05) pairwise difference	P value from Kruskal–Wallis
Pancreas	Autoimmune disease	589 (580 to 689)	AP + CP (p = 0.0006)	< 0.0001
	AP	731 (632 to 945)	No pancreas pathology (p = 0.0009) No disease (p = 0.0024)	
	AP + CP	1020 (897 to 1099)	Autoimmune disease (p = 0.0006) No pancreas pathology (p < 0.0001) No disease (p < 0.0001)	
	CP	700 (643 to 863)	No pancreas pathology (p < 0.0001) No disease (p = 0.0002)	
	No pancreas pathology	599 (560 to 649)	AP (p = 0.0009) AP + CP (p < 0.0001) CP (p = 0.0002)	
	No disease	583 (561 to 654)	AP (p = 0.0024) AP + CP (p < 0.0001) CP (p = 0.0013)	
Liver (5 categories)	Abnormal (steatotic)	631 (596 to 683)	N/A	0.179
	Abnormal (Other)	619 (559 to 740)		
	Isolated biliary dilation/prominence	592 (571 to 656)		
	No liver pathology	603 (584 to 664)		
	No disease	596 (577 to 648)		
Liver (3 categories)	Abnormal (steatotic)	631 (596 to 683)	N/A	0.0595
	Abnormal (Other)	619 (559 to 740)		
	Isolated biliary dilation/prominence + No Liver pathology + No disease	596 (578 to 662)		
Spleen	Abnormal	1215 (1102 to 1243)	N/A	0.396
	No spleen pathology	1178 (1129 to 1210)		
	No disease	1152 (1128 to 1207)		

*IQR* Interquartile range; *AP* acute pancreatitis; *AP* + *CP* acute on chronic pancreatitis; *CP* chronic pancreatitis

At 3 T, there were also significant differences in T1 relaxation time estimates between pancreas subgroups (p < 0.0001, Fig. 4 and Table 3). Significant pairwise differences (msec) were present between patients with no disease and patients with: acute pancreatitis (1087; 910 to 1259; p = 0.012), and acute + chronic pancreatitis (1226; 1025 to 1367; p < 0.0001) but not with chronic pancreatitis (942; 876 to 992 p = 0.680). T1 relaxation time estimates for patients with acute + chronic pancreatitis were significantly higher than for patients with chronic pancreatitis (p = 0.0245).

**Table 3:** Summary statistics for pancreas, liver and spleen T1 relaxation time estimates based on diagnostic grouping at 3 T. Pairwise comparisons were performed using Dunn’s test if Kruskal–Wallis test was statistically significant

Organ	Groupings	T1 relaxation time Median (IQR) [msec]	Significant (p < 0.05) pairwise comparisons	P value from Kruskal–Wallis
Pancreas	Autoimmune disease	784 (775 to 792)	None	< 0.0001
	AP	1087 (910 to 1259)	No pancreas pathology (p = 0.00195) No disease (p = 0.012)	
	AP + CP	1226 (1025 to 1367)	CP (p = 0.0245) No disease (p < 0.0001)	
	CP	942 (876 to 992)	AP + CP (p = 0.0245)	
	No pancreas pathology	827 (797 to 904)	AP (p = 0.0195)	
	No disease	779 (753 to 851)	AP (p = 0.0012) AP + CP (p < 0.0001)	
Liver (5 categories)	Abnormal (steatotic)	959 (848 to 997)	No disease (p = 0.0017)	0.0011
	Abnormal (Other)	882 (831 to 904)	No disease (p = 0.0455)	
	Isolated biliary dilation/prominence	950 (887 to 1012)	None	
	No liver pathology	836 (797 to 897)	None	
	No disease	818 (788 to 819)	Abnormal (Fatty) (p = 0.0017) Abnormal (Other) (p = 0.0455)	
Liver (3 categories)	Abnormal (steatotic)	959 (848 to 997)	Isolated biliary dilation/prominence + No liver pathology + No disease (p = 0.0028)	0.0023
	Abnormal (Other)	882 (831 to 904)	None	
	Isolated biliary dilation/prominence + No liver pathology + No disease	825 (797 to 887)	Abnormal (Fatty) (p = 0.0028)	
Spleen	Abnormal	1406 (1329 to 1464)	N/A	0.739
	No splenic disease	1390 (1290 to 1428)		
	No disease	1364 (1310 to 1433)		

*IQR* Interquartile range; *AP* acute pancreatitis; *AP* + *CP* acute on chronic pancreatitis; *CP* chronic pancreatitis

### Liver T1 relaxation time

Median T1 relaxation time estimates in patients with no liver disease was 596 (577 to 648) msec at 1.5 T and 818 (788 to 819) msec at 3 T. This was not significantly different (msec) from patients with no liver pathology (603; 584 to 664, p > 0.999 at 1.5 T and 836; 797 to 897, p > 0.999 at 3 T) or patients with isolated biliary dilation/prominence (592; 571 to 656 p > 0.999 at 1.5 T and 950; 887 to 1012, p = 0.224 at 3 T).

**Fig. 5:**
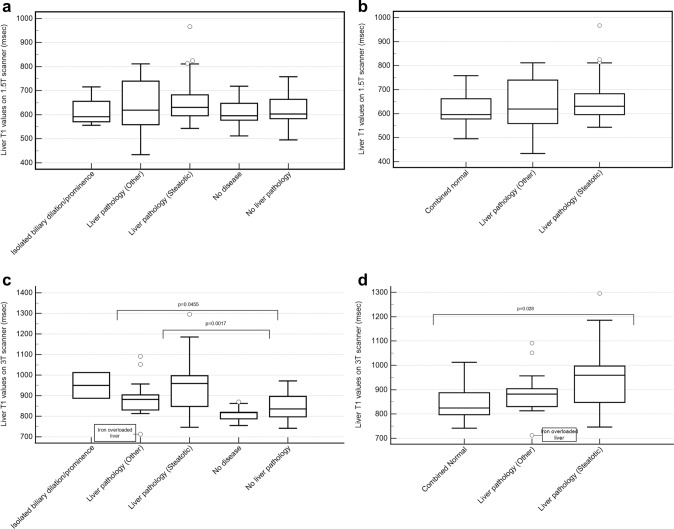
Tukey boxplots showing weighted (by region of interest size) T1 relaxation time estimates for liver (msec) based on **A** 5 categories [i.e., Abnormal (steatotic), Abnormal (Other), Isolated biliary dilation/prominence, No liver pathology, No disease (no liver or other pathologies)] on 1.5 T scanner, **B** 3 categories [i.e., Abnormal (steatotic), Abnormal (Other), Combined no disease (which includes Isolated biliary dilation/prominence No liver pathology, and No disease groups)] on 1.5 T scanner, **C** 5 categories on 3 T scanner, **D** 3 categories on 3 T scanner. Groupings were assigned according to scheme in Fig. 3. Circles represent outliers accompanied by their reasoning (if applicable). Significant pairwise comparisons (if present) between the no disease group and other groups have been shown with open brackets along with their respective p-value

When grouped into either 5 or 3 categories at 1.5 T, T1 relaxation time estimates were not significantly different among liver subgroups (p = 0.179 and p = 0.0595, Fig. 5 and Table 2).

When grouped into 5 categories at 3 T, T1 relaxation time estimates were significantly different among liver subgroups (p = 0.0011, Fig. 5 and Table 3). Pairwise differences (msec) were present between patients with no disease and: patients with steatosis (959; 848 to 997 p = 0.0017) and patients with other liver disease (882; 831 to 904, p = 0.0455). Similarly, when grouped into 3 categories, median T1 relaxation times were significantly different among groups (p = 0.0023) but with significant pairwise differences (msec) only between the combined no disease group (825; 797 to 887) and the steatosis group (p = 0.0028).

Liver T1 relaxation times were weakly correlated with liver PDFF at 1.5 T (r = 0.39, 95% CI: 0.24 to 0.52, p < 0.0001) and moderately correlated with liver PDFF at 3 T (r = 0.64, 95% CI: 0.49 to 0.76, p < 0.0001) (Supplementary Fig. 1).

### Spleen T1 relaxation time

Median T1 relaxation time estimates in patients with no disease was 1152 (1128 to 1207) msec at 1.5 T and 1364 (1310 to 1433) msec at 3 T. This was not significantly different (p > 0.999 for both) from patients without spleen disease at 1.5 T (1178; 1129 to 1210 ms) or 3 T (1390; 1290 to 1428 ms) (Supplementary Fig. 2 and Tables 2 and 3).

## Discussion

In our retrospective sample of children undergoing clinically indicated MRCP examinations, T1 relaxation times for the pancreas and liver are significantly different between patients without and with disease. There were no significant differences in spleen T1 based on the presence of disease. Patients with pancreatitis demonstrated longer T1 times than patients without pancreatic disease at both 1.5 T and 3 T. Patients with steatotic liver disease and patients with other liver diseases had longer T1 times than patients without disease at 3 T but not at 1.5 T. Our findings build on the body of literature, primarily in adults, that has shown differences in T1 relaxation times in patients with disease of the solid abdominal organs.

For the pancreas, Gilligan et al. previously reported mean T1 relaxation times in healthy children as 576 ms at 1.5 T and 730 ms at 3 T, similar to the medians of 583 ms and 779 ms at the same field strengths in our patients without disease [15]. To our knowledge, T1 relaxation times for children with pancreatic disease have not been reported. In adults, however, T1 relaxation times at 1.5 T were reported in one study as 726 ms for patients with mild chronic pancreatitis (Cambridge classification grade 2), similar to the 700 ms seen in our patients with chronic pancreatitis [11]. Adult studies at 3 T have also showed significantly longer T1 relaxation times in patients with chronic pancreatitis (1197 ms and 1099 ms) compared to their respective controls (840 ms and 797 ms) [18, 19] and significantly longer T1 relaxation times in patients with autoimmune pancreatitis (mean: 1124.5 ms) [12]. While these T1 relaxation times are higher than observed in our study for patients with chronic pancreatitis imaged at 3 T, they are similar to relaxation times for patients with acute and acute on chronic pancreatitis and for patients with no disease.

T1 mapping of the liver has been more extensively studied than T1 mapping of pancreas or spleen. The literature, however, includes studies employing either native T1 mapping or so-called corrected T1 (cT1) mapping which accounts for T2* effects associated with the presence of iron. Dillman et al. previously demonstrated that in children with autoimmune liver disease, T1 and cT1 are strongly correlated but with considerable bias between measurements indicating that T1 values from these two techniques cannot be directly compared. Among studies of T1 mapping, Gilligan et al. previously reported mean T1 relaxation times of 581 ms at 1.5 T and 738 ms at 3 T, similar to the median 596 ms at 1.5 T but lower than the median 818 ms at 3 T in our sample of children with no liver disease [15]. In a study of children with cardiac disease, but no assessment of liver disease, who were imaged at 1.5 T, Cho et al. reported a mean T1 relaxation time of 610 ms at 1.5 T, similar to the subgroup of patients without disease in our study.

T1 relaxation times, whether corrected or not, have been shown to be higher in patients with various liver diseases, including autoimmune liver disease, steatotic liver disease, and Fontan associated liver disease, among others. Increases in T1 relaxation times have been associated with fibrosis in adults with autoimmune hepatitis [13, 14, 20] and with liver fat content in patients with hepatic steatosis [21]. Our results show these expected elevations in T1 values in patients with steatotic or other liver diseases at 3 T but not 1.5 T. We did, however, observe a weak positive correlation between liver fat fraction and liver T1 relaxation time, an association that is similar to, but less strong than the same association previously shown in adults [21].

Previously reported spleen T1 relaxation times in patients without disease (1172 ms and 1356 ms at 1.5 T and 3 T respectively) are nearly identical to our sample. We did not find significant differences in spleen T1 relaxation times in patients with disease which is also in alignment with prior studies that showed no significant association between spleen T1 relaxation times and portal pressure measurements [13, 22].

Our study is limited by its retrospective design and convenience sample. Because of these factors, diagnoses and the absence of disease relevant to patient groups are derived and inferred from the medical record. Further, there is the potential that measured T1 relaxation times may be confounded by physiologic processes we have not accounted for including: fatty infiltration in the pancreas and elevated iron content in the liver and spleen. Relevant to acute pancreatitis, we did to capture the clinical severity of each episode of pancreatitis and therefore cannot explore how acute pancreatitis attack severity impacts T1 relaxation time.

## Conclusion

Pancreas T1 relaxation times are higher at 1.5 T and 3 T in children with pancreatitis and liver T1 relaxation times are higher in children with steatotic and non-steatotic chronic liver disease at 3 T. T1 relaxation time estimation has the potential to identify disease in solid organs and may have a future role as an imaging marker in children.

## Supplementary Information

Below is the link to the electronic supplementary material.

Supplementary file1 (DOCX 13 KB)
