# Supplementary material for: Magnetic resonance imaging T1 mapping of the liver, pancreas and spleen in children
Source: Abdom Radiol (NY). 2024 Jun 26;49(11):3963–74. doi: 10.1007/s00261-024-04428-z (PMC11519179; doi:10.1007/s00261-024-04428-z)
Supplement: Supplementary file 1 — Supplementary file1 (DOCX 13 KB) [file 261_2024_4428_MOESM1_ESM.docx]

**Supplementary Figure 1**: Scatterplots showing univariable correlations between Liver T1 relaxation time estimates (msec) and Liver proton density fat-fraction at (A) 1.5T and (B) 3T. Correlation strength was weak at 1.5T (r=0.39) and moderate at 3T (r=0.64)

**Supplementary Figure 2**: Tukey boxplots showing weighted (by region of interest size) T1 relaxation time estimates for spleen (msec) on (A) 1.5T scanner and (B) 3T scanner. Groupings are assigned according to the schema in Figure 3 and include Abnormal spleen finding, No spleen pathology and No disease (no spleen or other pathologies). Circles represent outliers and accompanied by their reasoning (if applicable).
